# Complete mitochondrial genome assembly and analysis of *Astragalus* (Papilionoideae: Fabaceae) species revealed its RNA editing and phylogenetic implications

**DOI:** 10.1186/s12870-026-08845-8

**Published:** 2026-04-29

**Authors:** Chunyu Tian, Yanting Yang, Wenlong Gong, Lemeng Liu, Zhiyong Li, Yumei Feng, Jianjiang Niu, Zinian Wu

**Affiliations:** 1 Institute of Grassland Research, Chinese Academy of Agricultural Sciences, Hohhot, China; 2 Key Laboratory of Grassland Resources and Utilization of Ministry of Agriculture, Hohhot, China

**Keywords:** *Astragalus*, Mitochondrial genome, Gene transfer, RNA editing, Phylogenetic analysis

## Abstract

**Background:**

*Astragalus* (Fabaceae), the largest genus of flowering plants with its phytogeographic center in Southwest Asia, is ecologically and medicinally vital, as high-quality forage and a source of traditional medicine. However, its ambiguous morphological traits hinder taxonomic clarity, prompting increased reliance on molecular phylogenetic approaches to resolve relationships.

**Result:**

In the research, we assembled and annotated mitochondrial genomes of three *Astragalus* species, integrating them with two published mitochondrial genomes for comparative analysis. Newly sequenced genomes (269,019–364,431 bp) encoded 55–59 genes (31–34 protein-coding, 22–25 tRNAs, 3 rRNAs). Comparative analysis identified four genes (*cox2*, *rps4*, *atp8*, *rps12*) with high nucleotide variability, establishing them as reliable molecular markers for *Astragalus* phylogenetics, critical for future taxonomic and evolutionary studies. Mitochondrial plastid sequences were widespread across all five species, with *petG* and *trnW-CCA* conserved as chloroplast-derived fragments. Phylogenetic trees based on concatenated mitochondrial genes revealed *Astragalus* species shared close affinities with *A. cicer* and *A.laxmanii*, refining subgeneric classification frameworks.

**Conclusion:**

Our study confirms mitochondrial genomes as powerful tools for resolving relationships in morphologically cryptic genera. The novel genomic resources, including repeats and RNA editing sites, and validated markers, lay a foundation for future research into *Astragalus* biogeography, adaptive evolution, and medicinal resource development—thereby, enhancing the genus’ ecological and economic value.

**Supplementary Information:**

The online version contains supplementary material available at 10.1186/s12870-026-08845-8.

## Introduction

*Astragalus* L., belonging to the Fabaceae family, is the largest genus of flowering plants, the species mostly distributed across temperate regions of the Northern hemisphere. Young shoots and leaves are not only used as forage but some dry roots of some species also can be used as an important traditional medicine, with almost 250 sections consisting of approximately 3,000 species distributed across all continents, except Australia [1]. In the southern and Sino-Himalayan regions of Asia, there are approximately 1,500–2,000 species, which are thought to be the diversity center of *Astragalus* [2]. *Astragalus* is widespread within the Old World and New World [3, 4] due to its different geographical origins, as well as complications in identification resulting from minor differences in morphology, which have led to phylogenetic controversy among many species. To date, extensive research has focused on identifying *Astragalus*, including morphological and molecular characteristic studies. Several researchers believe that adaptation to climatic change led to the convergence of vegetation and flowering, making identification difficult [2, 5]. The ancient hybridization phenomenon may be another factor that motivated speciation [6], posing a challenge for *Astragalus.*

With the development of sequencing technology, an increasing number of researchers have used molecular methods to interpret both inter-genus and intra-genus relationships in *Astragalus.* Based on the ITS sequences, researchers suggested that *A.* subg. *Pogonophace* is not a monophyletic group and have separated *Phyllolobium* from *A.* subg. *Pogonophace* [7]. Due to the characteristics of maternally inherited organisms, an increasing number of organelle genomes have been used for phylogenetic analyses. *Astragalus* was categorized into the Inverted Repeat-Lacking Clade along with *Oxytropis* and approximately 40 other genera of temperate herbaceous plants in the Astragalean, Vicioid, and Hedysaroid clades, plus the genera *Callerya*, *Wisteria*, and their close relatives, owing to the length of one inverted region [8–10]. Subsequently, chloroplast genome sequences of *matK*, *trnT-trnY*, *trnH-psbA* [11], *rpl32-trnL* [12], *trnL-F*, and *ndhF* [13, 14] were used to explore phylogenetic relationships at the molecular level.

Apart from the nuclear genome, all plants have two discrete organelles, chloroplasts and mitochondria, which contain their genetic materials [15]. The endosymbiotic theory is widely accepted [16]. The mitochondrial genomes originated from alpha-proteobacteria [17]. Compared to the chloroplast genome, mitochondria are more complicated and have a kaleidoscope structure, including linear, circular, multichromosomal, and other complex structures. To date, only 800 plant mitochondrial genomes are available, which is less than one-tenth of the chloroplast total(https://www.ncbi.nlm.nih.gov/nuccore, 2025-01-16). Given these complexities, it is not surprising that mitochondrial genome studies in large and complex genera like *Astragalus* are particularly lagging. As for *Astragalus*, there are 97 chloroplast genomes, while only the mitochondrial genomes of *A. membranaceus* [18] and *A. complanatus* can be retrieved from NCBI.

The substantial diversity of mitochondrial genome size, for example, *Silene conica* with 11.3 Mb, which is approximately 45.2 times the size of *Silene latifolia*, at 0.25 Mb [19, 20], together with considerable structural variability and the existence of appreciable gene fragment arrangement, as well as the inherent limitations of conventional technologies (extremely low throughput, difficulty in coping with large-sized and highly diverse genomes, uneven coverage that fails to fully capture repetitive sequences and structural variations, limited precision which makes it hard to distinguish gene fragment rearrangements from similar sequences, and cumbersome procedures that are incompatible with low-abundance and high-complexity samples), all hinder the advancement of plant mitochondrial genome research [21–23]. With the rapid development of next-generation sequencing (NGS), an increasing number of intricate plant mitochondrial genomes have been studied and interpreted in recent publications [24, 25].

Owing to the maternal inheritance characteristic of mitochondrial genomes, they are often used for phylogenetic analysis. Based on the reconstruction of phylogenetic trees covering approximately 75% of annual Astragalus species from the Old World and representatives of related perennial taxa, Nasim Azani et al. [8] proposed that geological events promoted the radiation of cushion-forming plants in adjacent mountainous regions. Other studies have shown that diurnal temperature variation may affect the biogeographical distribution of *Astragalus* species, but there is no clear correlation with their diversification processes [26].

To improve the understanding of *Astragalus* evolution and phylogeny and support fine variety breeding, we analyzed mitochondrial genome features and phylogenetic analysis of three assembled-annotated and two published *Astragalus* species. This study intends to clarify their intra- and interspecific phylogenetic relationships, offering insights into evolutionary history and novel directions for phylogenomics.

## Materials and methods

### DNA extraction, genome sequencing, and assembly

Fresh leaves of *A. melilotoides*, *A. cicer*, and *A. laxmannii*, identified by Professor Wu Zinian, the first author of the article, were collected from Hohhot, Inner Mongolia, China (40.57°N, 111.93°E). Plant samples were deposited at the National Perennial Forage Germplasm Resource Nursery (Hohhot, China; 40.57°N, 111.93°E). Genomic DNA was extracted from fresh leaves using the cetyltrimethylammonium bromide (CTAB) method and the Qiagen Blood & Cell Culture DNA Kit (Cat. no. 13323).

*Astragalus melilotoides* was sequenced using the Illumina MiSeq PE150 platform (Novogene Co., Ltd., Tianjin, China). Mitochondrial assembly was performed using the GetOrganelles software (V 1.7.5.3) [27] using default parameters (-R 50 -k 21,45,65,85,105,115 -P 1000000). The accuracy of the assembly results was further verified using Bandage visualization software [28] and mapping reads using Bowtie2 [29]. The raw reads of *A. melilotoides* from our previous study [30] were deposited in the NCBI Sequence Read Archive under accession number SRR13870430 (Table S1), and the complete mitochondrial genome sequence was deposited in GenBank (accession number OP279714).

*A. cicer* and *A. laxmannii* were sequenced by NGS Illumina Novaseq6000 combined with third-generation sequencing Nanopore PromethION (Illumina Novaseq6000 sequencing). To produce high-quality and accurate sequences by using Illumina short-read data to correct the inherent errors of Nanopore long-reads, then used for follow-up analyses. The nanopore data were then blasted against the reference sequences (plant mitochondrial core genes. https://github.com/xul962464/plant_mt_ref_gene) to obtain the mitochondria genome sequence. By using canu [31] to correct the data, NGS was then blasted to the corrected sequence using PMAT [32] and spliced to the third-generation sequencing data using Unicycler (V 0.4.8). Finally, Bandage (V 0.8.1) [28] software was used to visualize and manually adjust the stitching results.

The raw data of second and third-generation sequencing of *A. cicer* and *A. laxmannii* were deposited in NCBI Sequence Read Archive under accession number under the accession numbers SRR34934999, SRR34934998, SRR34934997 and SRR34934996, respectively (Table S1).

### Genome annotation

For the annotation of the three mitochondrial genomes, the protein codon genes (PCGs) and ribosomal RNA (rRNA) were annotated using Geseq software (V 2.03) [33] and PMGA (http://www.1kmpg.cn/pmga/) as *A. membranaceus* and *A. complanatus* references, followed by manual adjustment and correction using Geneious (V 2024.0.5) [34].

### Repeat sequence identification

The MISA software [35] was used to detect simple sequence repeats (SSR) in the four *Astragalus* mitochondrial genomes. Identification parameters included unit length (nucleotide)/min repeats: 1_10, 2_6, 3_4, 4_3, 5_3, and 6_3. Tandem Repeats Finder (TRF; V 4.09) [36] was used to probe tandem repeat sequences with specific parameter settings (2 7 7 80 10 50 2000 -f -d -m, corresponding to a match weight of 2, mismatch penalty of 7, indel penalty of 7, alignment score threshold of 80, minimum and maximum repeat unit lengths of 10 and 50 bp, respectively, maximum tandem repeat length of 2000 bp, with output of flanking sequences, detailed alignments, and unmasked low-complexity regions.). REPuter [37] was used to identify dispersed repeats exceeding 30 bp using a Hamming distance threshold of 3 and an E-value cutoff of 1 × 10^− 5^ to classify them as forward, reverse, palindromic, or complementary repeats.

### Codon usage

The values of the four *Astragalus* relative synonymous codon usage (RSCU) of the PCGs and the base composition of amino acids were calculated using CodonW 1.4.4. Perl scripts were used to perform codon preference to select unique coding sequences (CDS), and the results were plotted using R (V 4.4.1). The resulting RSCU patterns of every species single were visualized using an interactive RSCU plot generated with the RSCU-Plot Shiny app (https://pcg-lab.shinyapps.io/RSCU-Plot/) [38, 39].

### RNA editing

The RNA-editing sites of the PCGs in *A. melilotoides*, *A. cicer*, *A. laxmannii*, *A. complanatus*, and *A. membranaceus* were predicted using Deepred-mt [40], which has high accuracy due to the use of a convolutional neural network model. Results with a probability > 90% were retained.

### Mitochondrial plastid sequences

The assembly of the chloroplast genome *A. cicer* and *A. laxmannii* was performed by the software GetOrganelles (V 1.7.5.3) [27] using default parameters (Genbank accession numbers: PV423827, PV423828). *A. melilotoides*, *A. complanatus* and *A. membranaceus* chloroplast genomes deposited in GenBank (accession numbers: MW719855.1, NC_065023.1 and OR528897.1) (Table S2) were used as query sequences. The Advanced Circos Module of TBtools-II (V 2.136) [41] was used to research gene fragment transformation between mitochondrial and plastid genomes and display a circus map.

### Ka/Ks

Ka/Ks is the ratio of non-synonymous (Ka) to synonymous (Ks) substitution; a Ka/Ks value of 1 suggests neutral evolution, whereas values > 1 and < 1 suggest positive and purifying selection, respectively. To investigate evolutionary pressure, the MLWL method of Ka/Ks_Calculator (V 2.075) [42] was used to calculate the Ka/Ks of shared mitochondrial PCGs of *A. melilotoides*, *A. cicer*, *A. laxmannii*, *A. complanatus*, and *A. membranaceus*.

### Nucleotide diversity (Pi)

Pi is a significant indicator of a species’ population history. To clarify the evolution of each PCG, the software MAFFT (V 7.526) [43] was used to identify homologous gene sequences among diverse species, and DnaSP (V5.0) [44] was employed to calculate the Pi value corresponding to each gene.

### Synteny analysis of the mt sequences

BLASTN (V 2.13.0) [45] was used to compare sequences greater than 300 bp of five related *Astragalus* species pairwise (E-value: 1e^− 5^), and MCscanX [46] software with default parameters (match score ≥ 50, E-value ≤ 1e − 10, minimum syntenic block size ≥ 5 genes) was used to form and visualize a synteny picture to depict the relationship among the *Astragalus* species studied.

### Phylogenomic analysis

Mitochondrial genome phylogenetic analysis was performed based on 20 shared PCGs (*atp4*, *atp8*, *atp9*, *ccmB*, *ccmC*, *ccmFC*, *ccmFN*, *cob*, *cox3*, *matR*, *nad2*, *nad3*, *nad4*, *nad4L*, *nad5*, *nad6*, *nad7*, *nad9*, *rpl5*, *rps12*) and chloroplast genome phylogenetic analysis was performed based on 59 shared PCGs(*psbA*, *matK*, *rbcL*, *atpB*, *atpE*, *ndhC*, *ndhK*, *ndhJ*, *rps4*, *ycf3*, *psaA*, *psaB*, *rps14*, *psbZ*, *psbC*,* psbD*, *psbM*, *petN*, *rpoB*, *rpoC1*, *rpoC2*, *atpI*, *atpH*, *atpA*, *psbK*, *cemA*, *petA*, *psbJ*, *psbF*, *psbE*, *petG*,* psaJ*, *rps18*, *rpl20*, *psbB*, *psbN*, *psbH*, *petD*, *rpoA*, *rps11*, *rpl36*, *rps8*, *rpl14*, *rpl16*, *rps3*, *rps19*, *rpl2*, *rpl23*, *rps7*, *ndhF*, *ccsA*, *ndhD*, *psaC*, *ndhE*, *ndhG*, *ndhI*, *ndhA*, *ndhH*, *rps15*) derived from the species (Table S3) related to *Astragalus*, with *Oryza sativa* and *Triticum aestivum* chosen as outgroups to construct a phylogenetic tree. Chloroplast genome phylogenetic analysis based on the multiple sequence alignment MAFFT software [43] with default parameters. Mitochondrial phylogenomic analysis performed by two methods, maximum likelihood (ML) and Bayesian inference (BI), were used. Both the ML algorithm and Bayesian methods were used to construct the phylogenetic tree, and the best models were selected using ModelFinder [47]. Chloroplast genome and Mitochondrial genome phylogenetic ML analyses were conducted using RAxML (V 8.2.11) [48] with the GTRGAMMAI nucleotide substitution model, and node support was determined by bootstrap analysis with 1000 replicates. BI analyses were conducted using MrBayes (version 3.2.6) [49] with the GTR + F+I+G4 model. The parameters for the for the MrBayes analysis were set as follows: the number of generations was 2,000,000, the sampling frequency was 1,000, and the burn-in proportion was 25%.

## Results

### Genomic features of the three *Astragalus* mitogenomes

In the present study, three species, *A. melilotoides*, *A. cicer*, and *A. laxmannii*, were sequenced and annotated. The average coverage depth of sequencing for *A. melilotoides* using the Illumina MiSeq PE150 platform was 174.51×, yielding 28.56 million reads and 428 million bases. The average GC content was 37.29%, the percentage of bases with a quality score of at least 20 (Q20) was more than 96.98%, and the percentage of bases with a quality score of at least 30 (Q30) was more than 91.49% (Table S1, Figure S1). The average coverage depth of sequencing for *A. cicer* using NGS Illumina Novaseq6000 was 73.25× with 53.69 million reads and 1.61 billion bases. Its GC content was 37.12%, with Q20 > 97.90% and Q30 > 93.84%. For *A. laxmannii*, its coverage depth was 479.38×, generating 55.80 million reads and 1.69 billion bases for its mitogenomes (Table S1, Figure S2.1–S2.2). Its average GC content was 37.29, with Q20 > 96.98%, and Q30 > 91.49%. Nanopore sequencing for *A. cicer* yielded a coverage depth of 73.2x, producing 1.74 million reads and 1.63 billion bases. The mean read length was 9389 bp, and the N5f0 read length was 19,735 bp. However, *A. laxmannii* had 1.41 million reads and 1.59 billion bases, with an average coverage depth of 344.27× (Table S1, Figure S3.1–S3.2). The mean read length was 11,274 bp, and the N50 read length was 22,171 bp. The genome sizes of *A. melilotoides* and *A. cicer* were most similar, with one being 269,019 bp and the other 284,397 bp. *A. laxmannii’s was* 364,431 bp, greater than those of the two aforementioned species. However, GC content seemed unrelated to the length of the genome. The GC content of *A. cicer* was the highest, with a proportion of 45.45%, followed by *A. melilotoides* and *A. laxmannii*, which were 45.39% and 45.04%, respectively.

**Fig. 1:**
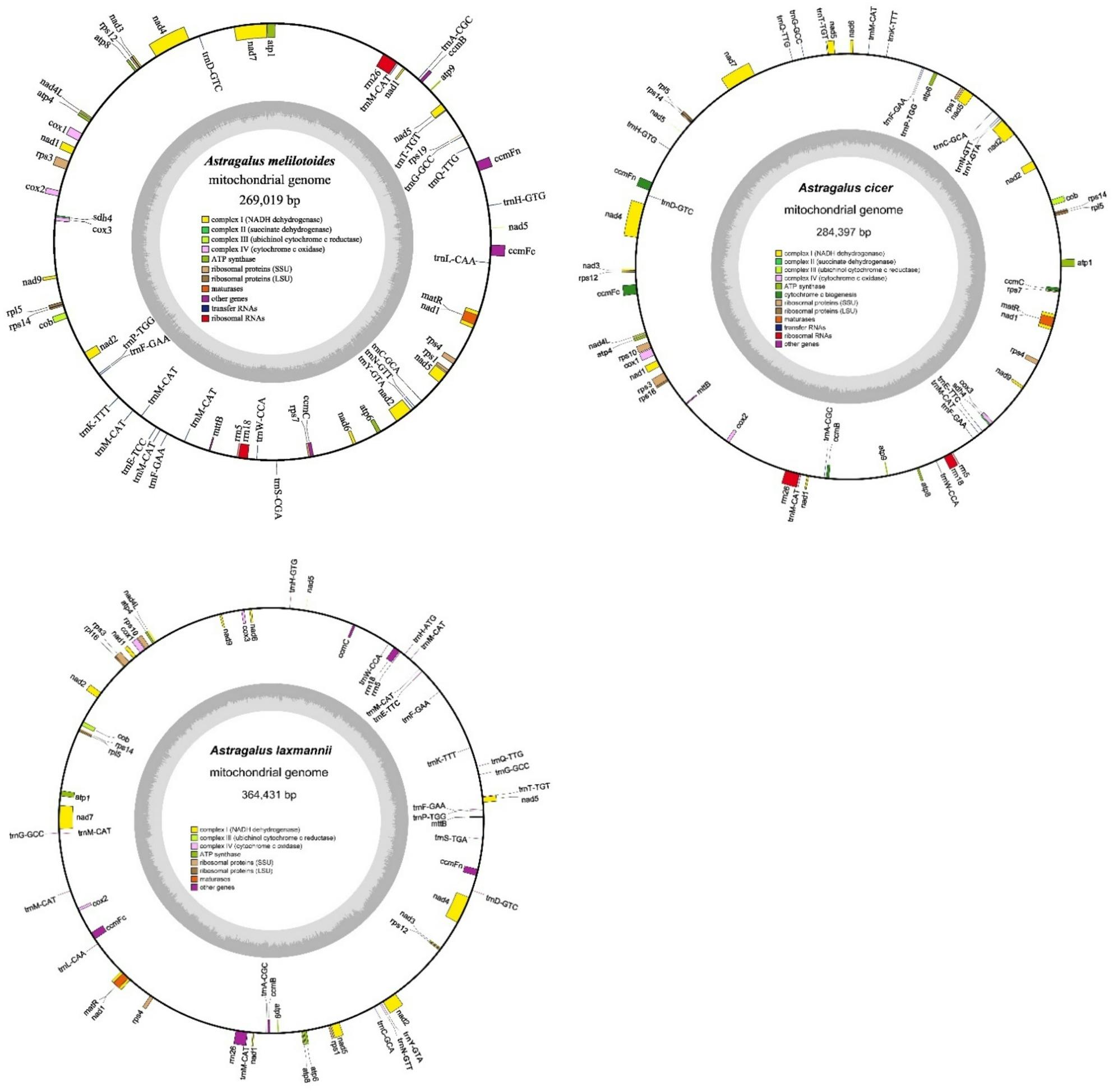
Circular map of the three *Astragalus* mitogenomes. Genes shown on the outside and inside of the circle are transcribed clockwise and counterclockwise, respectively. The dark gray region in the inner circle depicts GC content. Asterisks beside genes denote intron-containing genes. Genes are color-coded by functional category: yellow (NADH dehydrogenase subunits, Complex I); green (succinate dehydrogenase subunits, Complex II); brown (cytochrome bc₁ complex subunits, Complex III); purple (cytochrome c oxidase subunits, Complex IV); red (ATP synthase subunits, Complex V); cyan (cytochrome c maturation proteins); orange (large subunit ribosomal proteins); pink (small subunit ribosomal proteins); yellow squares (tRNAs with corresponding anticodons labeled). This map visualizes the genome structure, gene arrangement, and functional partitioning, serving as a foundational framework for subsequent comparative genomic and evolutionary analyses

The mitogenome size and the number of genes among different species was diverse. *A. laxmannii* had the largest genome (364,431 bp) and the gene count (59 genes) highest was well as *A. cicer*, consisting of 32 PCGs, 24 tRNA genes, and three rRNA genes, occupying 36,163 bp and accounting for 9.92% of the whole genome in *A. laxmannii*, followed by *A. melilotoides*, with 55 genes, occupying 13.08% (35,209 bp) of the whole mitogenome. All three species encoded three rRNA genes and two pseudogenes but had different tRNA genes. *A. cicer* had the fewest tRNA genes, totaling 18, while *A. melilotoides* had 22, a difference of four (Fig. 1).

The gene GC content, with minor differences among the three species, was 44.92%, 44.43%, and 44.74% in *A. melilotoides*, *A. cicer*, and *A. laxmannii* mitochondria, respectively. In the *A. laxmannii* mitogenome, the GC content was 43.07%, 51.52%, and 51.95% in PCGs, tRNA, and rRNA genes, respectively. However, in the *A. cicer* mitogenome, the tRNA genes had the highest GC content at 51.66%, followed by the GC content of rRNA genes and PCGs, which were 51.79% and 42.87%, respectively. The trend in the GC content of genes in the *A. melilotoides* mitogenome was the same as in *A. laxmannii*, with rRNA genes having the highest GC content (51.77%), followed by tRNA (51.43%) and PCGs (43.14%).

Compared with *A. cicer*, the ribosomal protein gene *rpl5* had only one copy in the mitogenomes of *A. melilotoides* and *A. laxmannii.* Furthermore, *A. melilotoides* lacked *rpl16.* As far as cytochrome c oxidase genes are concerned, *cox1* and *cox2* are general genes in the three species; *cox3*, *cox4*, and *cox5* were unique to the mitogenomes of *A. melilotoides*,* A. cicer*, and *A. laxmannii*, respectively. The ribosomal protein genes differed among the three species. *A. cicer* had two copies of *rps14*, one more than *A. laxmannii* and *A. melilotoides*, but *rps19* existed in *the A. melilotoides* mitogenome, which lacked *rps10* compared with the other species. The tRNA genes showed significant interspecies differences. Compared with *A. melilotoides*, *A. cicer* not only lacked the gene *trnL-CAA* and *trnS-CGA* but also lacked two copies of *trnM-CAT.* Furthermore, *trnG-GCC* had two copies, one more than *A. melilotoides* and *A. cicer*. *trnH-ATG* and *trnS-TGA* were specific to the *A. laxmannii* mitogenome (Table 1, Table S4–S7).

**Table 1:** Gene annotation in the *Astragalus* mitogenome

Group of genes	Gene name			
	A. melilotoides	A.cicer	A. laxmannii	A. complanatus
ATP synthase	*atp1*,*atp4*,*atp6*,*atp8*,*atp9*	*atp1*,*atp4*,*atp6*,*atp8*,*atp9*	*atp1*,*atp4*,*atp6*,*atp8*,*atp9*	*atp1*,*atp4*,*atp6*,*atp8*,*atp9*
Cytohrome c biogenesis	*ccmB*,* ccmC*, ccmFc*,* ccmFn*	*ccmB*,* ccmC*, ccmFc*,* ccmFn*	*ccmB*,* ccmC*, ccmFc*,* ccmFn*	*ccmB*,* ccmC*, ccmFc*,* ccmFn*
Ubiquinol cytochrome c reductase	*cob*	*cob*	*cob*	*cob*
Cytochrome c oxidase	*cox1*,*cox2*,*cox3*	*cox1*,*cox2*,*cox4*	*cox1*,*cox2*,*cox5*	*cox1*,*cox2*,*cox3*
Maturases	*matR*	*matR*	*matR*	*matR*
Transport membrane protein	*mttB*	*-*	*mttB*	*mttB*
NADH dehydrogenase	nad1****,nad2****,*nad3*,nad4***,*nad4L*, nad5****,*nad6*,nad7****,*nad9*	nad1****, nad2****,* nad3*, nad4***,* nad4L*, nad5****,* nad6*, nad7****,* nad9*	nad1****, nad2****,* nad3*, nad4***,* nad4L*, nad5****,* nad6*, nad7****,* nad9*	nad1****, nad2****,* nad3(2)*, nad4***,* nad4L*, nad5****,* nad6*, nad7****,* nad9*
Ribosomal proteins (LSU)	*rpl5*	*rpl16*,* rpl5(2)*	*rpl16*,* rpl5*	*rpl16*,* rpl5*
Ribosomal proteins (SSU)	*#rps7*,*rps1*,*rps12*,*rps14*,*rps19*,*rps3*,*rps4*	*#rps7*,* rps1*, rps10*,* rps12*,* rps14(2)*,* rps3*,* rps4*	*#rps7*,* rps1*, rps10*,* rps12*,* rps14*,* rps3*,* rps4*	*#rps7*,* rps1*, rps10*,* rps12(2)*,* rps14*,* rps3*,* rps4*
Succinate dehydrogenase	*#sdh4*	*#sdh4*	*#sdh4*	*#sdh4*
Ribosomal RNAs	*rrn18*,*rrn26*,*rrn5*	*rrn18*,*rrn26*,*rrn5*	*rrn18*,*rrn26*,*rrn5*	*rrn18*,*rrn26*,*rrn5*
Transfer RNAs	*trnA-CGC*,* trnC-GCA*,* trnD-GTC*,* trnE-TTC*,* trnF-GAA(2)*,*trnG-GCC*,* trnH-GTG*,* trnK-TTT*,* trnL-CAA*,* trnM-CAT(5)*,*trnN-GTT*,* trnP-TGG*,* trnQ-TTG*,* trnS-CGA*, trnT-TGT*,*trnW-CCA*,* trnY-GTA*	*trnA-CGC*,* trnC-GCA*,* trnD-GTC*,* trnE-TTC*,* trnF-GAA(2)*,* trnG-GCC*,* trnH-GTG*,* trnK-TTT*,* trnM-CAT(3)*,* trnN-GTT*,* trnP-TGG*,* trnQ-TTG*, trnT-TGT*,* trnW-CCA*,* trnY-GTA*	*trnA-CGC*,* trnC-GCA*,* trnD-GTC*,* trnE-TTC*,* trnF-GAA(2)*,* trnG-GCC(2)*,* trnH-ATG*,* trnH-GTG*,* trnK-TTT*,* trnL-CAA*,* trnM-CAT(5)*,* trnN-GTT*,* trnP-TGG*,* trnQ-TTG*,* trnS-TGA*, trnT-TGT*,* trnW-CCA*,* trnY-GTA*	*trnC-GCA*,* trnD-GTC*,* trnE-TTC(2)*,* trnF-GAA(2)*,* trnG-GCC(2)*,* trnH-GTG*,* trnI-CAT(2)*,* trnK-TTT*,* trnL-CAA*,* trnM-CAT(3)*,* trnN-GTT*,* trnP-TGG*,* trnQ-TTG*, trnQ-TTG*,* trnS-CGA*, trnT-GGT*,* trnW-CCA*,* trnY-GTA*,* trnfM*

*:the numbers of * indicates the introns number

#Gene: Pseudo gene

Gene(X): the numbers in parentheses indicates the number of copies of multi-copy genes

The lengths of most PCGs were conserved across the species studied. However, notable differences were observed in some genes, particularly in *A. complanatus*. Of these 31 PCGs, 16 PCGs were of the same length. However, the differences in length between *A. laxmannii* and *A. cicer*, even *A. melilotoides*, were small, but *A. complanatus* was greater than the above three species. In addition, the same RNA-editing site was detected in the stop codons of *ccmFc* and *nad4L* in all four species. Notably, in the remaining PCGs, there were only four genes, *mttB*, *nad5*, *rpl5*, and *rps3*, whose lengths were the longest in the *A. complanatus* mitogenome among the four species. In particular, *mttB* was 146 bp longer than that of the other three species. Compared to *A. melilotoides*, *A. cicer*, and *A. laxmannii*, an RNA-editing site in the stop codon of atp6 was unique to *A. complanatus*, which transformed the codon from CAA to TAA. Similarly, *cox2* had editing sites in its start codon only in *A. complanatus.* In contrast, *nad1* possessed RNA editing in its start codon, which changed the codon from ACG to ATG, but the same gene was absent in *A. complanatus.* The gene *nad5* also lacked RNA-editing sites in the stop codon of the *A. complanatus* mitogenome compared with the other three species.

### Repetitive sequences

**Fig. 2:**
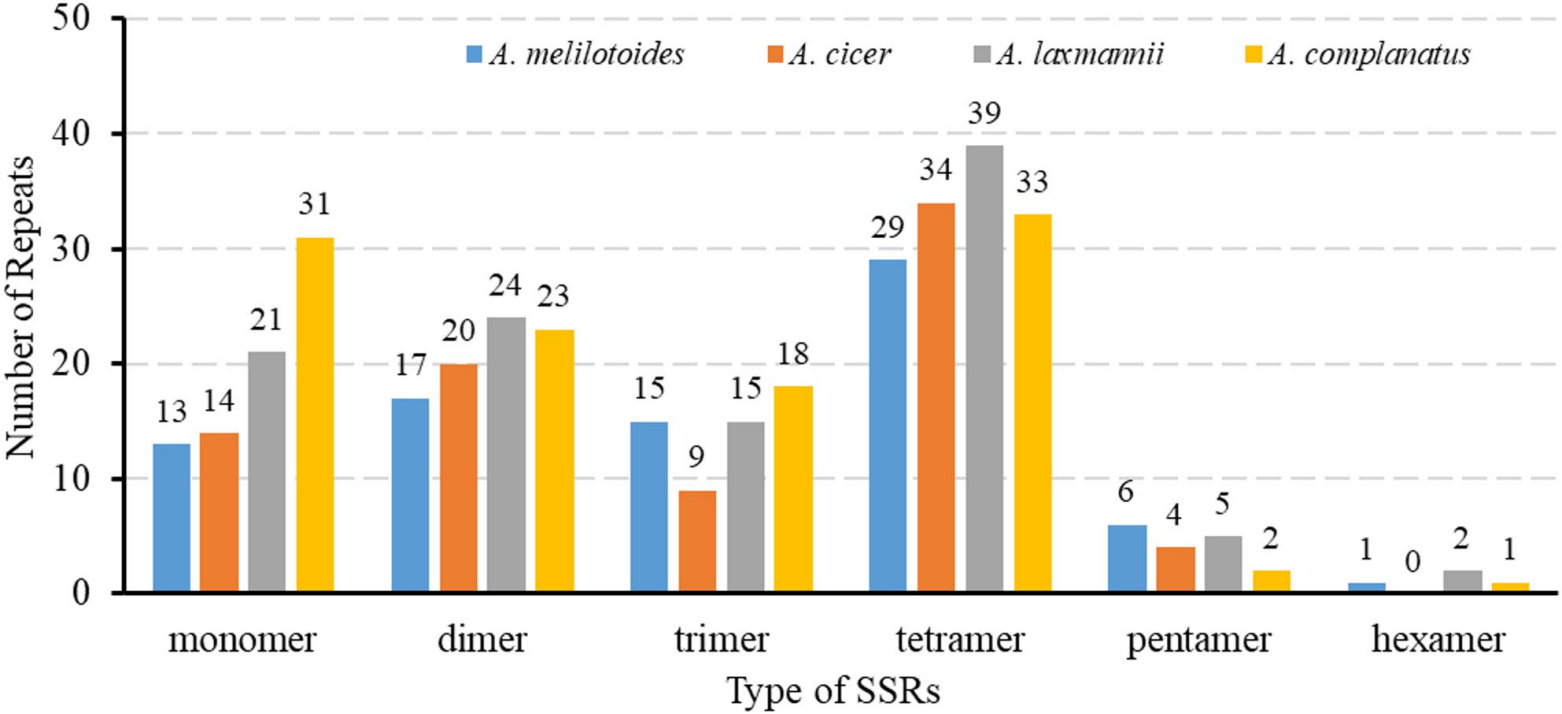
Distribution of simple sequence repeats (SSRs) in the four *Astragalus* mitochondrial genomes

Repetitive sequences perform important functions in gene expression, transcriptional regulation, and chromosome arrangement. In minor studies, SSR dispersed repeats have been analyzed. *A. complanatus* had the highest number of SSRs (108), followed by *A. melilotoides*, *A. cicer*, and *A. laxmannii.* There were 106 SSRs in the *A. laxmannii* genome, *A. melilotoides* and *A. cicer* had the same number (81). Regardless of the species, tetramers were most abundant, whereas hexamers were infrequent. In particular, no hexamers were found in the *A. cicer* mitogenome. Concerning the type of repeat unit, *A. cicer*, *A. laxmannii*, as well and *A. complanatus*, had the same tendency, from more to less frequent tetramers, dimers, monomers, trimers, pentamers, and hexamers. In the *A. melilotoides* mitogenome, the number of trimers was higher than that of the monomers (Fig. 2). Among the monomer repeats, *A. cicer* and *A. laxmannii* contained A/T bases. In the *A. melilotoides* and *A. complanatus* mitogenomes, A/T bases exceeded C/G bases, accounting for 84.62% and 87.10%, respectively (Table S8). Because *A. membranaceus* has been depicted in previously published research [18], its SSRs are only listed in the supplementary material (Table S8), which was used for comparison and to obtain an intuitive understanding of all public *Astragalus* species. Similarly, we no longer describe the characteristics that have been analyzed before in the following chapter, and the relevant contents are listed in the supplementary table.

Regardless of the species, most SSRs were distributed in the intergenic spacer (IGS) region, and only a few SSRs are located in gene or intron regions. In *A. melilotoides*, *A. cicer*, and *A. complanatus*, SSRs are found in 12 gene or intron regions, but only eight of these have SSRs in the *A. laxmannii* and *A. membranaceus* mitogenomes. Among them, the introns of the NADH dehydrogenase gene comprise *nad1*, *nad2*, *nad4*, *nad5*, and *nad7.* However, the *ccmFC* intron, which contains SSRs, was detected only in *A. complanatus* and *A. membranaceus* mitogenomes. In five *Astragalus* species, the gene *rps3* and the intron of *nad4* all had an SSR distribution (Tables S8–S9).

**Fig. 3:**
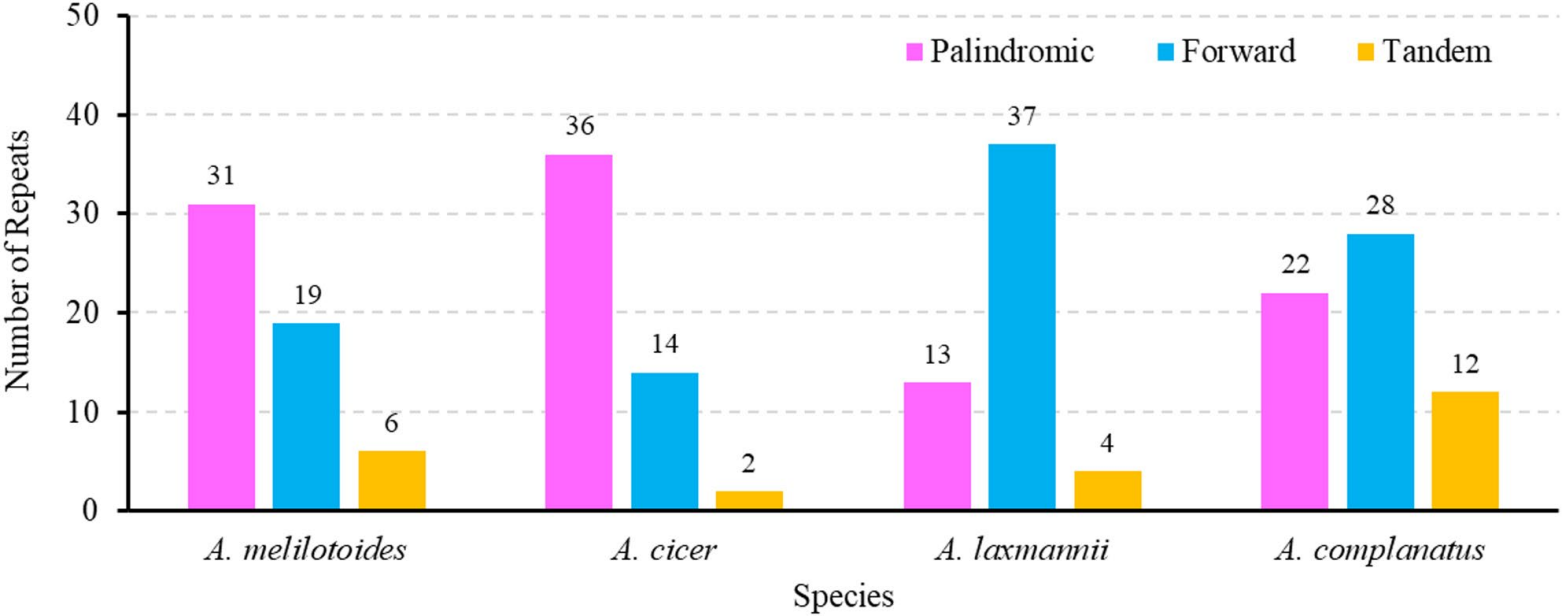
Allocation of the lengths of dispersed repeats in the four *Astragalus* mitochondrial genomes. The X-axis indicates the types of species, and the ordinate indicates the number of scattered and tandem repeats

Dispersed repeats are another important type of repeat distributed in the mitochondrial genome. No reverse repeats existed. Compared with the other three species, there were more dispersed repeats in *A. complanatus*. In the *A. complanatus* mitochondrial genome, 62 dispersed repeats were identified—six, eight, and eight more than in *A. melilotoides*, *A. cicer*, and *A. laxmannii*, respectively. Palindromic repeats were the most abundant in both *A. melilotoides* and *A. cicer* mitogenomes, followed by forward and tandem repeats. They had the greatest number of forward repeats, followed by palindromic and tandem repeats. Among the four species, *A. cicer* had the most palindromic repeats; forward repeats were most abundant in *A. laxmannii*, and tandem repeats were more abundant in the *A. complanatus* mitochondrial genome than in the other three species (Fig. 3, Table S10).

The locations of the dispersed repeats are complex and varied. Most dispersed repeats were located in two IGS regions, each IGS formatted by two genes. Just a few dispersed repeats whose one part was located in the genes and the other in IGS regions, or one part in introns of the genes and the other in IGS regions; in addition, there were also repeats situated in two genes or different fragments of the same gene. Additionally, diversity was also observed as far as the IGS region concerned. Most IGSs were the intergenic spacers of two different genes; some were the intergenic spacers of genes and gene exons or gene introns. Three dispersed repeats, whose two parts of them are located in *trnM-CAU* in the mitogenome of *A. melilotoides*, the same phenomenon was also detected in *A. laxmannii* mitogenomes, which were different from the *A. melilotoides*, there were only two dispersed repeats located in the *trnM-CAU* in the *A. laxmannii* mitogenome. *In the A. cicer* mitogenome, one dispersed repeat is divided into five parts: *rpl5*, *cob*, *rps14*, *rps14*, and r*pl5* (Table S10).

**Fig. 4:**
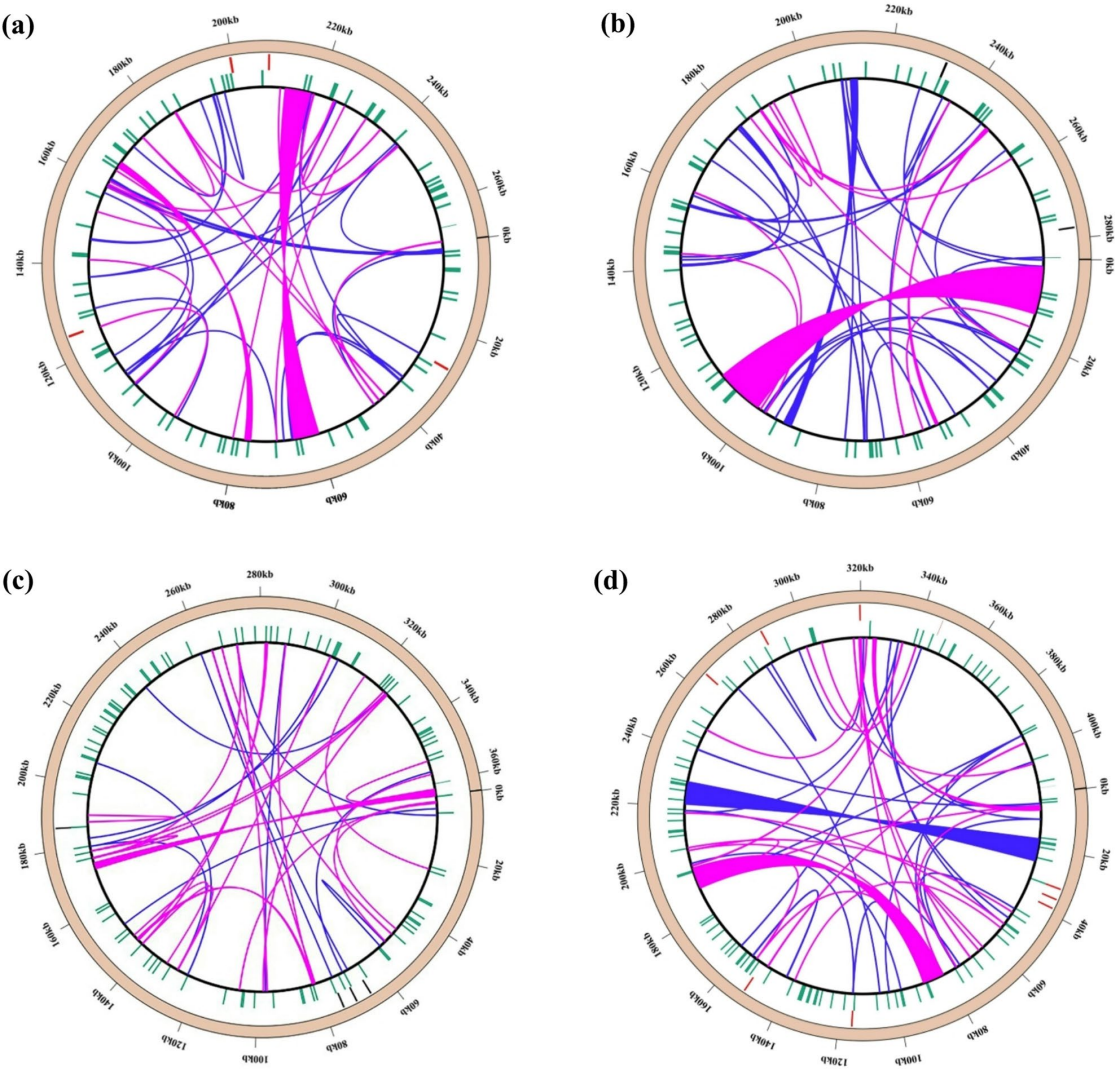
Repeat analysis of the mitochondrial genome in four *Astragalus*. **a** *A. melilotoides*, (**b**) *A. cicer*, (**c**) *A. laxmannii*, (**d**) *A. complanatus.* The inner circle shows the forward repeats and palindromic connected with fuchsia and blue links, respectively. The next circles show the tandem (black) repeats and microsatellites (green) as short bars. The interval scale was 20 kb

Dispersed repeat analysis showed that only four repeats were located in PCGs of *A. melilotoides* mitogenome, with others in IGSs across species. Repeats in *A. melilotoides*, *A. cicer*, and *A. laxmannii* were 8–19 bp, while *A. complanatus* harbored four long repeats (> 30 bp, including 63-bp and 96-bp). Most tandem repeats were in IGSs, with only four in *A. melilotoides* (*nad5* and *cox2*, three in *cox2*; Fig. 4, Tables S10, S11).

### Codon usage analysis of PCGs

**Fig. 5:**
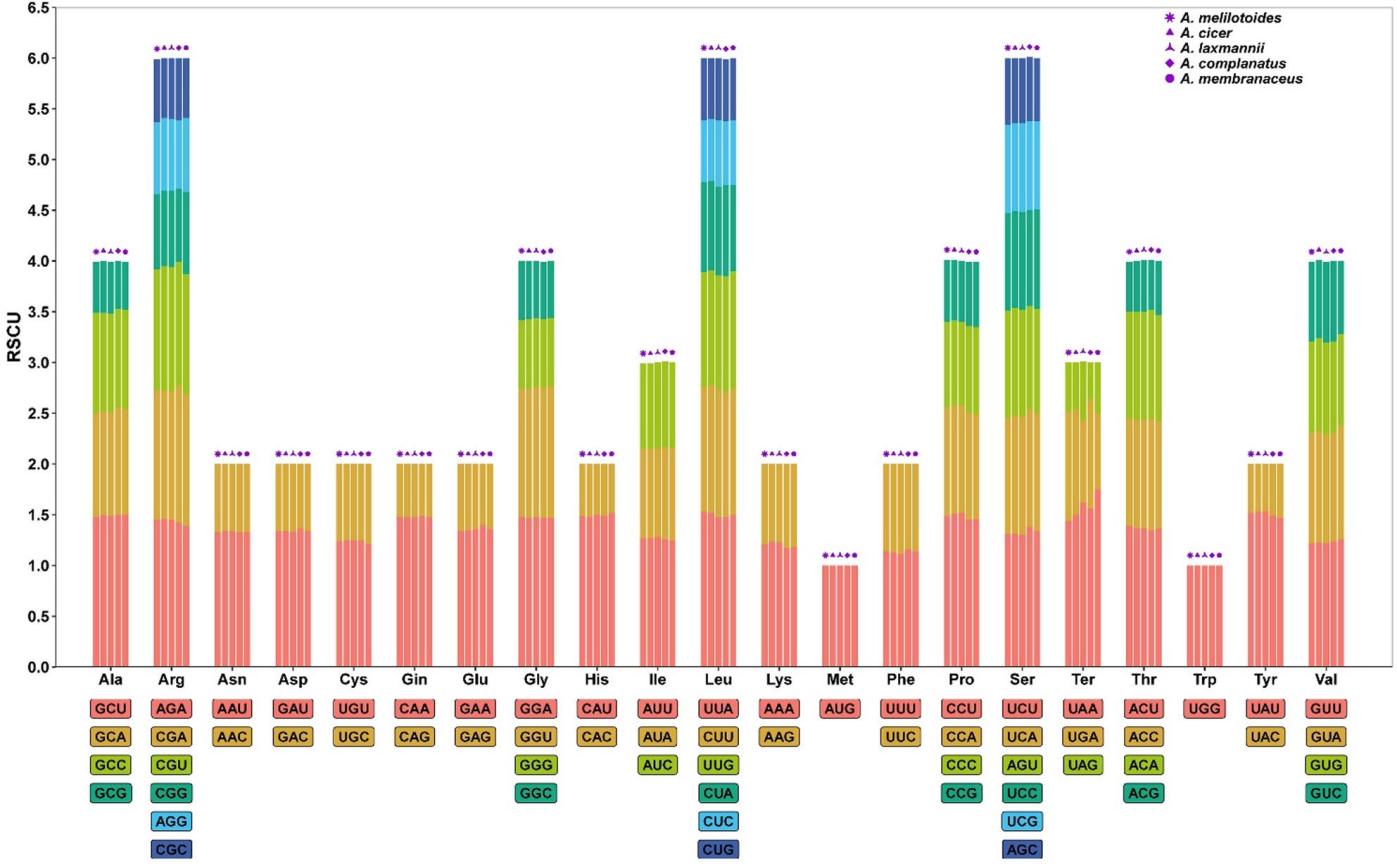
RSCU of five *Astragalus* mitogenome. Codon families are on the X-axis. RSCU values are the number of times of a particular codon, relative to the number of times that the codon would be observed for a uniform synonymous codon usage

According to the codon usage analysis of the PCGs, 64 codons encoding 21 amino acids were identified. In *A. melilotoides*, *A. cicer*, and *A. complanatus*, 32 identical optimal codons encoded 19 amino acids. However, *A. laxmannii* and *A. membranaceus* mitogenome both lacked UGA compared to the three species above and had 31 optimal codons. Among the five species, the start codons AUG and UGG encode methionine (Met) and tryptophan (Trp), respectively, with RSCU values of 1, and no strong bias was observed. Although the values of RSCU are different, the stop codon UAA is the most optimal in *A. laxmannii*,* A. complanatus*, and *A. membranaceus* mitochondrial genomes. In *A. melilotoides* and *A. cicer* genomes, the most optimal codons were UUA, encoding leucine (Leu), and UAU, encoding tyrosine (Tyr), with a value of 1.53 (Fig. 5, Table S12). Besides, RSCU of five species was also performed (Figure S4).

### RNA editing

**Fig. 6:**
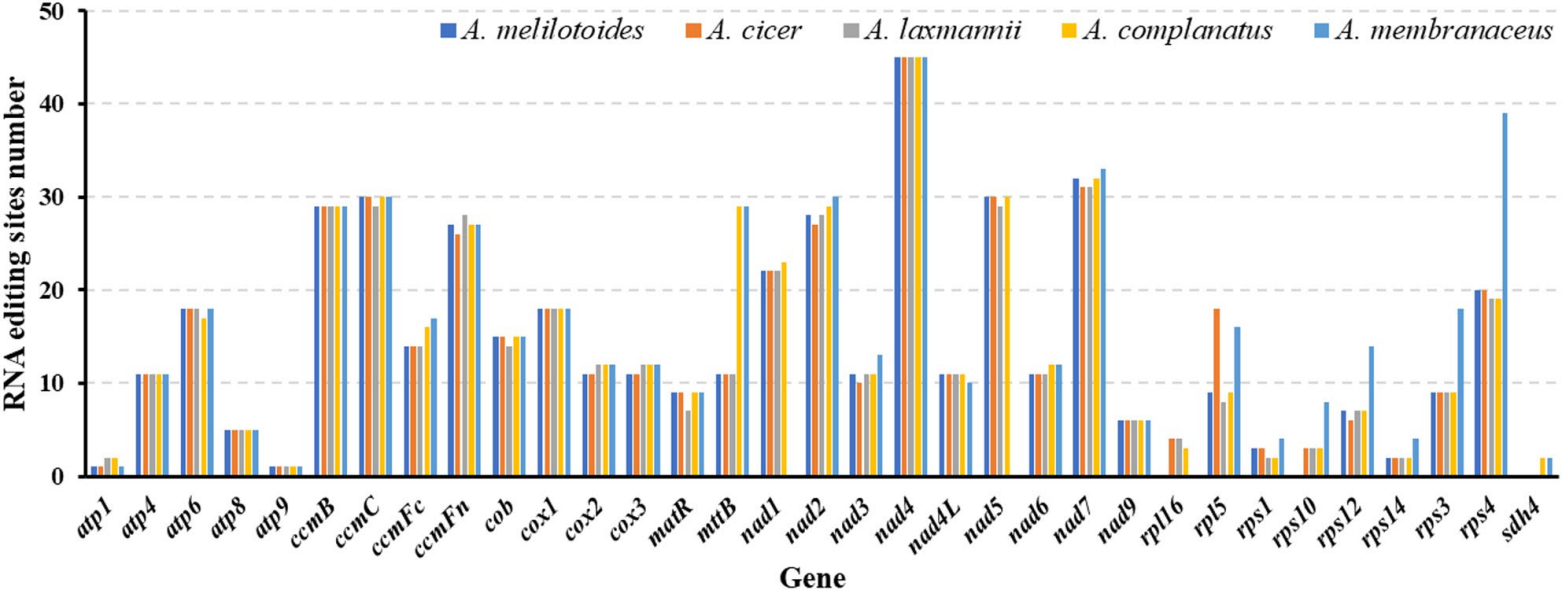
The distribution of RNA-editing sites in mitogenome protein-coding genes of five *Astragalus* species

RNA editing was a widespread phenomenon in the five *Astragalus* species, affecting between 30 and 34 protein-coding genes in their mitogenomes. According to RNA editing, *A. complanatus* acquired five start codons, followed by *A. melilotoides*, *A. cicer*, *A. laxmannii*, which had four start codons, and *A. membranaceus* with three start codons. Regardless of the species, *cox2* obtained two start codons and *nad7* gained one. In terms of stop codons, *A. complanatus* had none in its mitogenome, and *A. melilotoides*, *A. cicer*, *A. laxmannii*, and *A. membranaceus* had one and two stop codons, respectively. In all the studied species, *dispersed repeats* with 45 sites and the gene with the most RNA-editing sites were observed. In contrast, the gene *atp1* merely had only one site. Compared with each other, genes *atp4* and *nad1*, *nad5* lacked RNA-editing sites in *A. membranaceus*,* rps10* without RNA-editing sites in *A. melilotoides*, *rpl16* without RNA-editing sites both in *A. melilotoides* and *A. membranaceus* mitochondrial genomes, nevertheless, gene *sdh4* just had RNA-editing sites in *A. complanatus* and *A. membranaceus* genomes. Regardless, the editing sites all occurred at the C-base and were transformed into the U-base. Among all RNA-editing sites, the most conversion took place at the first site of the codon (59–64%), followed by the second site (30–35%), and only about 4% occurred at the third site (Fig. 6, Tables S13–S17).

**Fig. 7:**
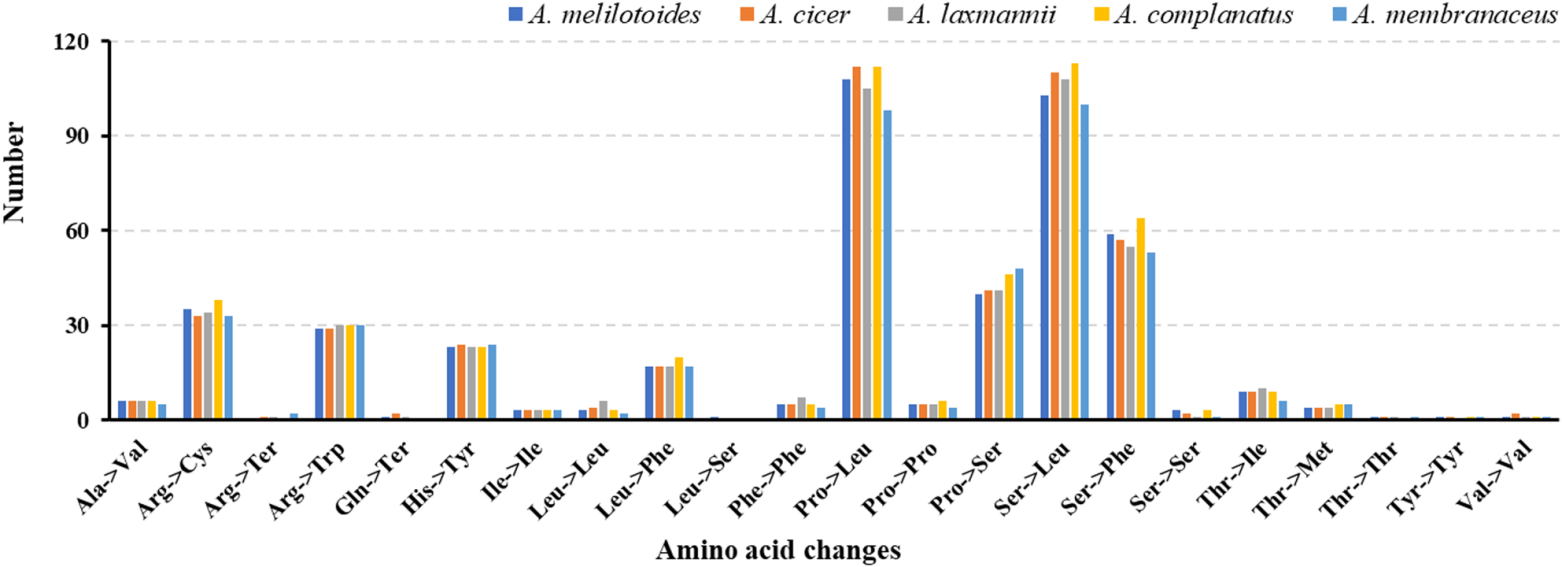
Frequency of amino acid changes caused by RNA editing in four *Astragalus* species

Because of RNA editing, many amino acids were changed. Except for *A. complanatus*, one, two, three, and three hydrophilic amino acids transformed into terminators in the mitogenomes of *A. melilotoides*, *A. laxmannii*, *A. cicer*, and *A. membranaceus*. In studied *Astragalus* species, proline (Pro) was changed to leucine (Leu), serine (Ser) to leucine, serine to phenylalanine (Phe), proline to serine, and arginine (Arg) to cysteine (Cys). Conversely, arginine to terminator, glutamine (Gln) to terminator, and leucine to serine ratios were comparatively small. Although RNA editing exists, there are several synonymous phenomena such as leucine to leucine, phenylalanine to phenylalanine, proline to proline, serine to serine, threonine (Thr) to threonine, tyrosine (Tyr) to tyrosine, and valine (Val) to valine. Based on the classification of hydrophilicity and hydrophobicity, the proportion of hydrophilic amino acids that transformed into hydrophobic amino acids was the highest. In *A. membranaceus*, 222 amino acids were converted from hydrophilicity to hydrophobicity, followed by *A. complanatus*, *A. cicer*, *A. laxmannii*, and *A. melilotoides*; the amounts of conversion were 221, 209, 207, and 204, respectively. Some hydrophobic amino acids transformed to hydrophilicity were also observed. Among *A. melilotoides*, *A. cicer*, and *A. laxmannii* genomes, 41 amino acids transformed to hydrophilicity, and 46 and 52 amino acids that changed from hydrophobicity transformed to hydrophilicity in *A. complanatus* and *A. membranaceus* mitochondrial genomes, respectively. In all five species, the types of amino acid transformations were hydrophilic to hydrophobic, hydrophobic to hydrophobic, hydrophilic to hydrophilic, and hydrophobic to hydrophilic (Fig. 7, Tables S13–S17).

### Mitochondrial plastid sequences (MTPTs)

DNA can be transferred between the nucleus and organelles or intra-organelle genomes. The gene fragments derived from the chloroplast (cp.) genome that were transferred to the mitochondrial genome are known as MTPTs. According to the homologous fragments identified within the mitochondrial and chloroplast genomes, there were 23, 25, 34, 36, and 15 MTPTs in *A. melilotoides*, *A. cicer*, *A. laxmannii*, *A. complanatus*, and *A. membranaceus*, respectively. Except in *A. membranaceus*, the total size of all homologous sequences in the cp. genome was greater than that in the mitogenome. Among these, the difference in homologous sequences was the greatest, and the fragments in the cp. genomes were 118 bp longer than those in the *A. laxmannii* mitogenome. Conversely, the differences in *A. cicer* were minimal, and the gene sequences in the mitogenome were seven base pairs shorter than those in the cp. genome. The homologous sequences comprised 4.9% of the *A. laxmannii* mitogenome and accounted for the highest proportion, followed by *A. complanatus*,* A. melilotoides*,* A. cicer*, and *A. membranaceus*, with ratios of 3.7%, 1.9%, 1.8%, and 1.7%, respectively. The most homologous fragments were located in the IGS in the mitogenomes of four species: *A. cicer*, *A. laxmannii*, *A. complanatus*, and *A. membranaceus*. Merely one fragment contained two genes, *trnM-CAU* and *trnG-GCC*, in the mitogenomes of the five species. In addition to *A. melilotoides* and *A. cicer*, partial sequences of two homologous fragments were situated in introns in the cp. genomes of the other three species.

**Fig. 8:**
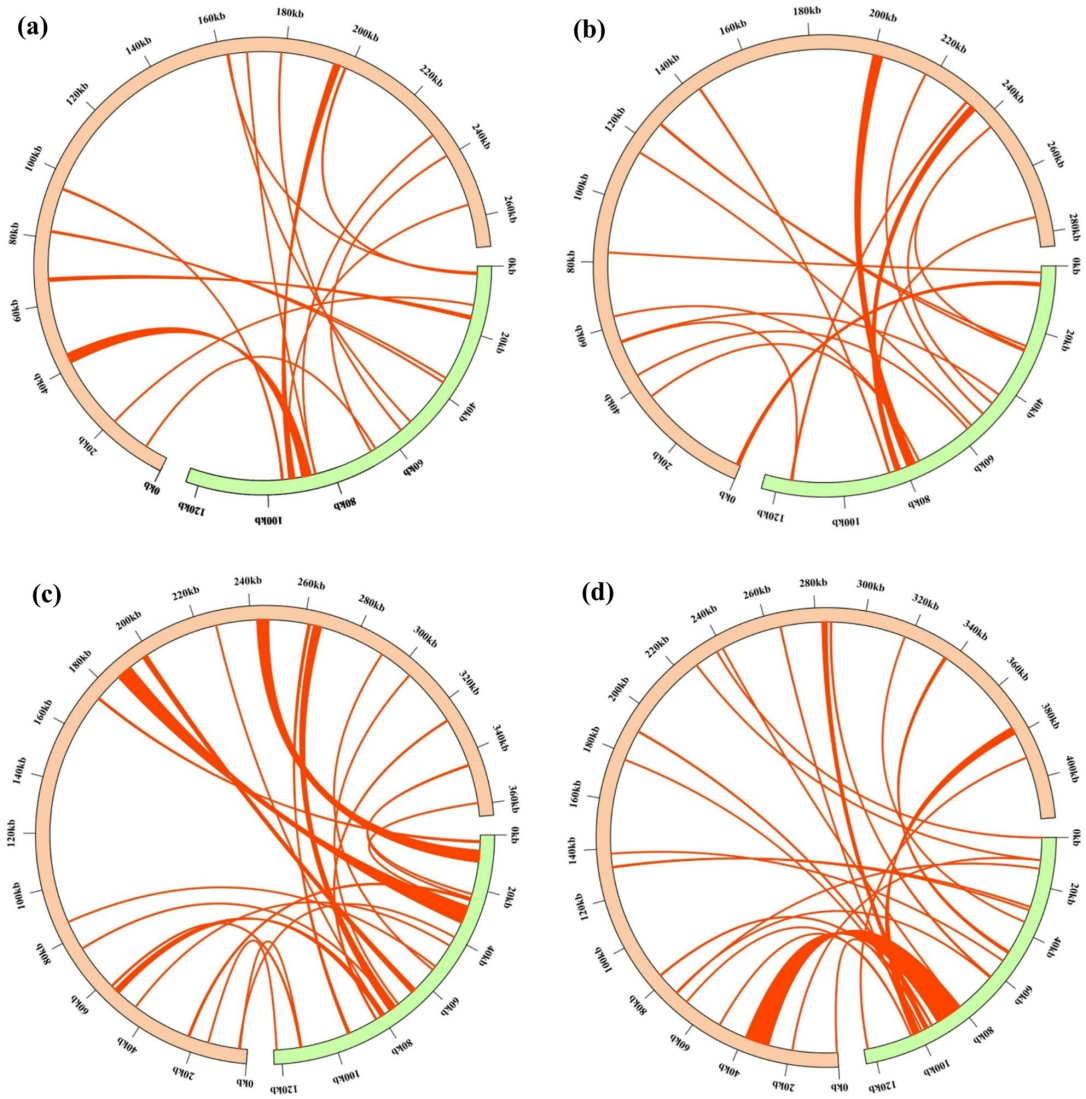
DNA transfer between the chloroplast (light lime green) and mitochondrial (light salmon) genomes. **a** *A. melilotoides*, (**b**) *A. cicer*, (**c**) *A. laxmannii*, (**d**) *A. complanatus.* Dots and heat maps inside the two chromosomes show where genes are located. The orange lines in the circle show the regions of chloroplast-like sequences inserted from the chloroplast genome into the mitochondrial genome

However, among the five *Astragalus* species, homologous fragments in the cp. genome occurred mostly in rRNA and tRNA, and only a few were found in PCGs and IGSs. One fragment was located in five genes: *trnG-GCC*, *trnM-CAU*, *psaA*, *psaB*, and *rps14*. Moreover, another two fragments occupied three genes, including *rps2*, *rpoC2*, and *atpI*, as well as the intron of *ndhA*, *ndhH*, *ndhA* in the cp. genome of *A. laxmannii*. Notably, regarding *petG* and *trnW-CCA* as a whole, it was a homologous fragment in all five species of the cp. genome, and the corresponding MTPTs in the other four species all contained *trnW-CCA* except for *A. complanatus*, which were located in IGS of *ccmFn* and *nad6* (Fig. 8, Table S17).

### Comparison of the mitogenome among 40 fabaceae related species

**Fig. 9:**
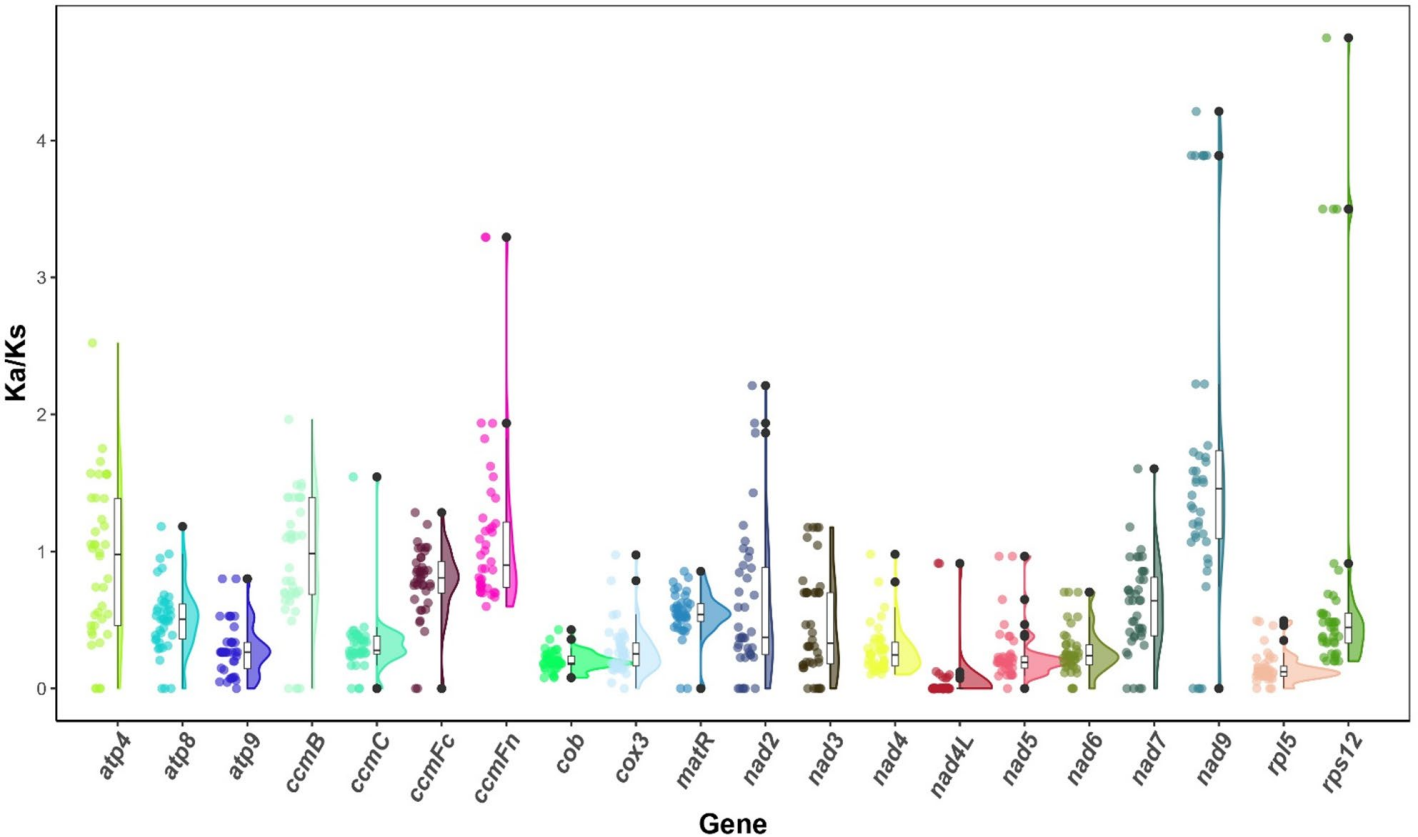
Ka/Ks values of 20 shared genes in mitogenomes of *A. cicer* versus 40 Fabaceae species

Ka/Ks is an indicator of the evolution of nucleic acid molecules. This ratio can be used to infer whether there is selective pressure acting on the PCGs. The ratio could produce three outcomes: Ka/Ks > 1 indicates that the gene is under positive selection, Ka/Ks = 1 indicates neutral selection, and Ka/Ks < 1 suggests negative selection. By comparing 20 shared genes in the mitogenomes of *A. cicer* with 40 Fabaceae species, it was found that for nine genes—*rpl5*, *cob*,* nad5*, *nad6*, *nad4*, *nad4L*, *atp9*, *cox3*, *matR—*the ratio of Ka/Ks was no more than 1, and all had negative selection. There was no neutral selection of PCGs in any of the 40 Fabaceae species. Compared with other genes, the gene *nad9* showed the strongest signal of positive selection, being under positive selection in 33 species where it was analyzed. The genes *nad2*, *nad7*, *nad3*, *rps12*, *ccmFc*, and *atp8*, in just several species, with no more than ten, experienced positive selection. In particular, for *atp8*, the value of Ka/Ks exceeding 1 was detected only in *Vicia faba*, one species. *A. laxmannii*, *Lotus japonicus*, *Glycine max*, and *Glycine soja* showed only one gene that was exposed to positive selection. For a single species, there were at most five genes with Ka/Ks > 1 (Fig. 9; Table S19).

### Nucleotide diversity

**Fig. 10:**
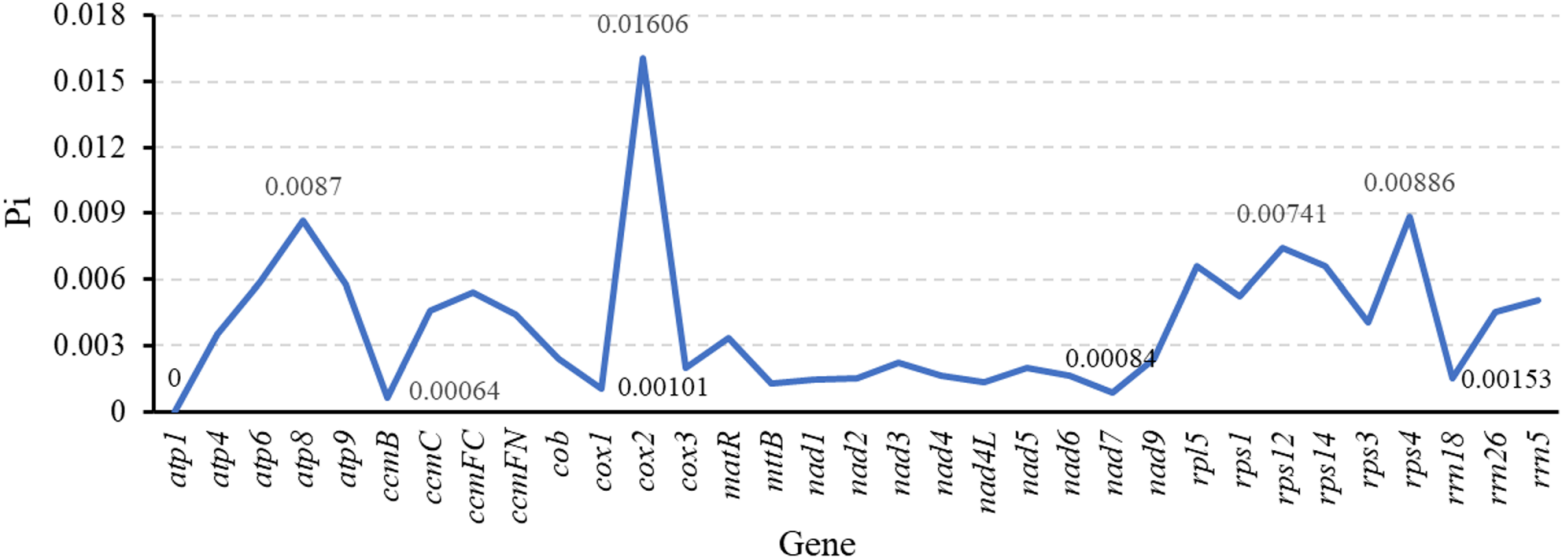
Nucleotide diversity of five *Astragalus* species (30 shared genes)

Nucleotide diversity (Pi) serves as an essential measure for evaluating variation within nucleic acid sequences among different species and regions with high variation can be used as molecular markers in population genetics studies. Based on the analysis of 30 shared genes among the five *Astragalus* species, the Pi value ranged from 0 to 0.01606. Only the Pi value of gene *cox2* was more than 0.1 and was the highest, followed by *rps4*, *atp8*, *rps12*, etc. (Fig. 10, Table S20).

### Synteny analysis of the mt sequences

**Fig. 11:**
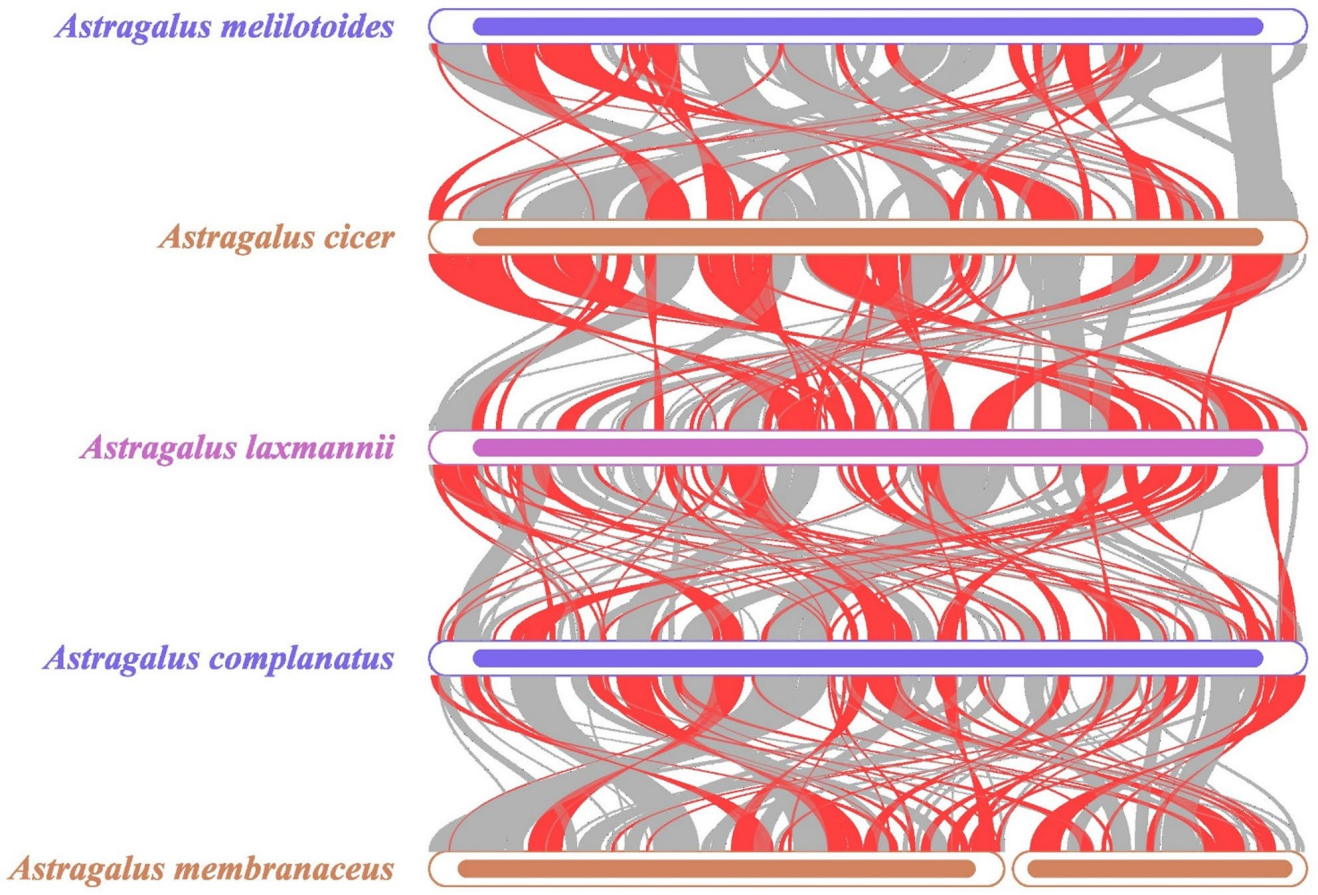
The mitochondrial genomes of *A. melilotoides*, *A. cicer*, *A. laxmannii*, *A. complanatus*, and *A. membranaceus* are arranged from top to bottom. Red-curved regions represent inversion events, gray regions indicate highly conserved syntenic blocks with strong sequence homology, and white regions correspond to species-specific sequences. This visualization reveals the dynamic structural evolution of *Astragalus* mitogenomes

To infer relationships among the five *Astragalus* species, the BLASTN program was utilized to analyze homologous genes and sequence arrangements. The size of homologous blocks in the *A. complanatus* mitochondrial genome was the longest compared with the other four species, then followed by the homologous blocks in *A. membranaceus*, *A. laxmannii*, *A. cicer*, and *A. melilotoides*. As a reference genome, the proportion of homologous blocks in the mitogenome of *A. cicer* was the highest, up to 83.05%. However, the proportion of homologous blocks in the mitogenome of *A. membranaceus* was highest in the query genome. According to the results, there were massive rearrangements in the homologous interspecies blocks. In addition, some inversions occurred among different species, indicating that gene recombination occurred during species evolution (Fig. 11; Table S21 ~ Table 25).

### Phylogenomic analysis

**Fig. 12:**
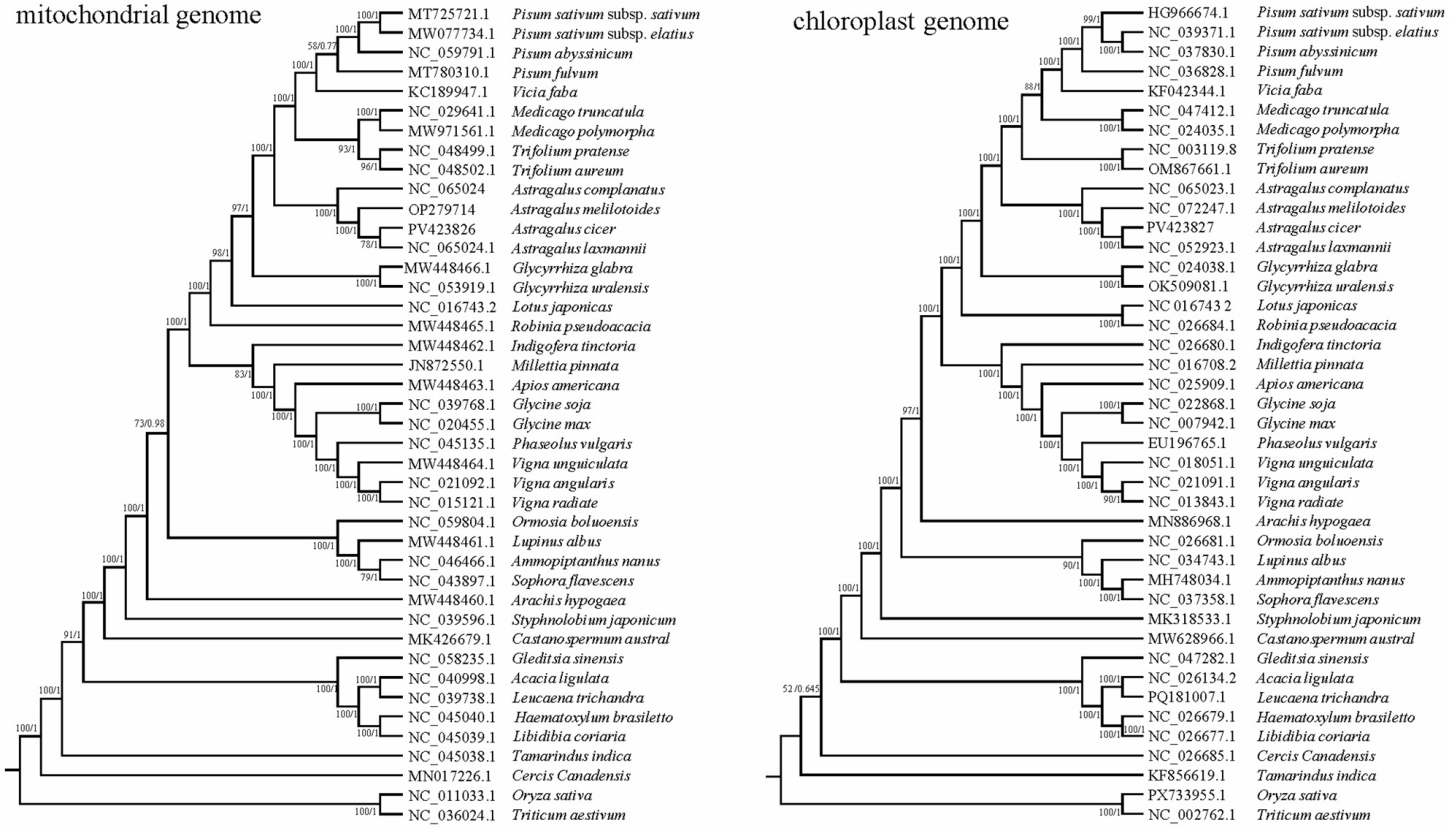
Phylogenetic tree of four *Astragalus* with other 36 represented Fabaceae species. *Oryza sativa* and *Triticum aestivum* were selected as the outgroups. The maximum likelihood and Bayesian tree were constructed based on 20 and 59 shared protein-coding genes in mitogenome (left) and chloroplast (right) phylogenomic analysis, respectively. Maximum likelihood (ML) bootstrap support values/Bayesian posterior probabilities are shown for each node

Due to its low substitution rate and maternal inheritance, the organelle genome is typically used for phylogenetic analyses. In this study, 20 shared PCGs of mitochondrial genomes and 59 shared PCGs of mitogenomes in the five studied *Astragalus* species, 36 representing Fabaceae species, along with two outgroups, *O. sativa* and *T. aestivum*, were chosen for evolutionary analysis. Overview two phylogenomic tree, most of the maximum likelihood bootstrap support values/bayesian posterior probabilities nearly one, which indicated that the phylogenetic relationships are well supported. However, some outcomes of cluster were different among two phylogenomic analysis. No matter mitogenomes tree or cp. genomes, among the studied species, *Cercis canadensis* of Caesalpinioideae was the closest to the root of the phylogenetic tree. *A. complanatus* was also closest to the phylogenetic tree root formed by the *Astragalus* species. *Astragalus* species clustered in a monophyletic group and had the closest relationship with two clades: one was formed by four *Pisum* species along with *Vicia faba*, and the other clade was formed by four species, consisting of two *Medicago* and two *Trifolium* species. However, there also some differences between two phylogenomic tree, *Pisum abyssinicum* evolved firstly than the clade formed by *Pisum sativum* subsp. *sativum* and *Pisum sativum* subsp. *elatius.* But in the cp. genome phylogenomic analysis, *Pisum sativum* subsp. *sativum* dependent with the *Pisum sativum* subsp. *elatius* and *Pisum abyssinicum.* In addition, the lacation of *Ormosia boluoensis* has significant difference. In mitochondrial phylogenetic tree, *Arachis hypogaea* diverged earlier than clade formed by *Ormosia boluoensis*, *Lupinus albus*, *Ammopiptanthus nanus*, and *Sophora flavescens*, however, the results were opposite with it in the phylogenomic analysis formed by chloroplast genomes. The same situation was also existed between *Tamarindus indica* and *Cercis Canadensis* (Fig. 12).

## Discussion

### Mitochondrial genome structure and size variations

Mitochondria are indispensable eukaryotic organelles that undertake oxidative phosphorylation and cellular energy supply, maintain intracellular calcium homeostasis, and play a crucial role in regulating programmed cell death. With advances in technology, an increasing number of phylogenetic analyses have been performed on organelle genomes. The mitochondrial genome has undergone more frequent architectural variations, leading to gene loss, transfer, structural alteration, and rearrangement, compared to chloroplasts and their counterparts in animals [25]. Additional factors, such as the existence of long repeats, MTPTs, and nuclear - mitochondrial transfer fragments (NUMTs) [47], hinder the development of the mitochondrial comparative genomic studies. To date, only one Astragalus species, A. membranaceus, has had its mitogenome analyzed and described in research [47]. The lengths of the three Astragalus species sequenced or annotated ranged from 269,019 to 364,431 bp with GC content ranging from 45.04% to 45.45%. Due to the presence of two circular chromosomes in the genome, the size difference between A. membranaceus and the three species mentioned above was significant. However, the GC content was similar to that of A. membranaceus at 45.30%. In contrast to previous studies that relied mainly on short-read sequencing, we applied a hybrid strategy combining Oxford Nanopore long-read sequencing and Illumina short-read polishing, which enabled us to generate a complete, high-contiguity mitochondrial genome assembly with a validated double-ring structure. This pipeline significantly improved assembly accuracy, structural resolution, and base-level correctness compared with assemblies based solely on second-generation sequencing data.

Although the genome size may be significantly larger than others, the type and amount of mitochondrial genome coding were similar. These were divided into three types: PCGs, rRNA genes, and tRNA genes. Correspondingly, the researched *Astragalus* species also had three kinds of genes [48]. However, gene loss, gain, and copy number variation are still observed in these species. All five species had lost *rps2*, *rps11*, and *rps13*. According to the researches, three genes transferred to the nuclear genomes more likely which led to the loss in mitigmomes [49]. However, the pseudogene *rps7* existed in four species compared to other Fabaceae species [50], resulted in that incompletely transfer to the nuclear and occurred dependently [51]. In addition to *nad2*, *nad4*, and *nad7*, which have more than one intron, similar to *Dalbergia odorifera* [52] and *Trigonella foenum-graecum* [53] in Fabaceae, the remaining NADH dehydrogenase genes have several introns, including additional *nad3*, *nad4L*, and *nad9*. The evident differences prioritized tRNAs. *A. cicer* had the least type of gene or copies. For instance, there were only three copies of *trnM-CAT*, whereas there were five copies of *trnM-CAT* in the other three *Astragalus* species tested. The *trnM-CAT* gene, an essential tRNA gene, showed considerable copy number variation among species. There were one, three, four, five, five, and five copies of it in *D. odorifera*, *A. cicer*, *T. foenum-graecum*, *A. melilotoides*, *A. laxmannii*, and *A. complanatus*, respectively, which may contribute to genomic rearrangement and adaptive evolution [54], besides, it might cause the differences of mitochondrial translation efficiency [55]. The amount and number of copies of the gene are partial causes of interspecies differences [52, 53].

### Characteristics of the repeats

The rearrangement induced by repeat DNA is a significant factor in the architectural modification of mitogenomes [56]. Among the five species studied, there were two main types of repeats: SSRs and dispersed repeats. According to previous research, the majority of dispersed repeat sequences are located in the IGS, which belongs to non-coding regions. Recombination led by the rearrangement of the repeat sequence could give rise to the insertion and appearance of novel ORFs, which produce novel genes correspondingly [57], in addition to affecting genetic transcription, alteration of gene expression, or gene repositioning [58, 59]. The presence of repeats in *Astragalus* species generates a series of consequences, such as homologous recombination, ectopic homologous recombination, highly variable structural organization [60], and there is still a need for extensive research.

### Analysis of codon usage

In addition to the stop codons, 61 triplets or codons correspond to 20 amino acids. Because the numbers of codons and amino acids are not equivalent, most amino acids are usually encoded by two to six synonymous codons. Codon usage bias is a phenomenon wherein synonymous codons are used at different frequencies and exists extensively in viruses, animals, and plants. When one or more codons are translated more frequently in the translation process, they are preferred. Similar to *Vigna umbellata* [61] in the Fabaceae, AUG and UGG showed no usage bias for the five *Astragalus* species. UAA was the optimal codon for *A. laxmannii*,* A. complanatus*, and *A. membranaceus*, whereas in *A. melilotoides and A. cicer* mitogenomes, the optimal codons were UUA and UAU, respectively. The difference in the most optimal codons may be because the forces they experience from selection, mutation, and genetic drift were different [62], but the mechanism remains unclear.

### Prediction of RNA editing

With evolution, various mitochondrial genetic code phenomena, such as trans-splicing and RNA editing, have arisen [63]. RNA editing affects a range of organisms, including viruses, animals, and plants [64]. It can involve enzymatic base transitions, modifications, deletions, or insertions [56]. Three types of RNA editing have been reported: nucleotide substitutions, post-transcriptional insertions or deletions, and co-transcriptional insertions or deletions. In the case of *Astragalus* species, RNA editing involves nucleotide substitutions, with amounts ranging from 456 to 488, similar to other Fabaceae species [52, 53]. There are also some consistent and puzzling phenomena; *nad4* has the most RNA-editing sites in different species belonging to various families, often unrelated [53, 60, 64], possibly related to the content of introns. Regardless of the position of the codon, RNA editing involved all conversions from C to U, mainly occurring at the second codon position. RNA editing changes the hydrophobicity and hydrophilicity of proteins by altering their amino acid sequences. In this study, the transformation of proteins from hydrophilicity to hydrophobicity was the most common, followed by other transformations such as hydrophobicity to hydrophilicity. The transformations between hydrophobicity and hydrophilicity can alter the protein structure, functional stability, and behavior in organisms [65], However, its effects on protein function and ecological adaptability of *Astragalus* membranaceus need to be further analyzed. Hydrophilic/hydrophobic conversion may regulate key stress resistance processes such as ion transport, osmotic adjustment or redox balance by changing the stability of the transmembrane domain, the microenvironment of the active site or the protein interaction interface [66, 67]. Combined with the ecological characteristics of drought and salt tolerance of *A. membranaceus*, it is speculated that these editing events may be involved in the fine regulation of stress response [68]. Unfortunately, this study did not perform functional verification or structural prediction, resulting in a logical fault between the phenomenon description and the ecological conclusion, which needs to be compensated by combining the stress response analysis of editing efficiency and protein structure simulation. What’s more, the loss of RNA edit might directly affect the assembly and function of the respiratory chain complexes in plant mitochondria. In maize, When the editing function of *DEK618* on the four genes *cob-298*, *ccmC-184*, *ccmB-428*, and *ccmFC-1219* is completely abolished, the assembly and function of mitochondrial complex II are impaired, resulting in abnormal mitochondrial morphology [69]. Loss of RNA editing at nad1, nad3, and nad7 mediated by PPR767 leads to reduced complex I activity, ROS accumulation, and disrupted mitochondrial structure of rice [70]. However, whether *Astragalus* species physiological activities and functions are affected by changes in protein characteristics requires further exploration.

### Chloroplast-derived mitogenomic sequences

Genetic material can be transferred among the nucleus, mitochondria, and chloroplasts within the cell [71]. Notably, MTPT can occur in unrelated species through horizontal gene transfer [72]. MTPT events were first detected in maize [73], and they comprised 1% to 10% of the higher plant mitochondrial genomes [74]. Among the five *Astragalus* species, the proportion of MTPT was 4.9%, 3.7%, 1.9%, 1.8%, and 1.7% in the mitogenomes of *A. laxmannii*, *A. complanatus*,* A. melilotoides*,* A. cicer*, and *A. membranaceus*, respectively. Among all five species, only one sequence was located in the tRNA genes *trnM-CAU* and *trnG-GCC*; the others were situated in the IGS or the intron of the different genes, which corroborates the theory that MTPTs were almost nonfunctional and that the tRNAs were replaced with the equivalents from the chloroplasts [75].

### Analysis of Ka/Ks ratios and Pi

Because of their characteristic simple and intuitive expression, Ka/Ks or dN/dS are often used to indicate the evolutionary pressures on the PCGs, depending on their different values. However, Ka/Ks changes with differences in the population sample [76], and the amino acid differences between the two orthologous proteins are also affected by the time elapsed since their common ancestor [65]. In different species, the degree of evolutionary pressure on the same PCGs varies. The Ka/Ks value of *rps12* in *A. laxmannii* was the highest at 4.75. Regardless of the species, the ratio of Ka/Ks of most shared PCGs was less than 1, which indicates that most non-synonymous are deleterious and are therefore removed by purifying selection [77]. Pi is another significant indicator in population genetics or population genomics research. In many species, the genes *cox2*, *rps12*,* and nad7* all had a high Pi value [78], indicating that these genes have the potential to develop into molecular markers. Ka/Ks, along with nucleotide diversity, showed that *Astragalus* mitochondrial genomes have a slow evolutionary rate. Nevertheless, some homologous fragment rearrangements were observed in all five species (Fig. 12).

### Phylogenetic inference

Owing to their uniparental inheritance and slow evolutionary rates, organelle genomes, consisting of chloroplast and mitochondrial genomes, have become effective tools for researching plant taxonomy. With the advent of sequencing technology, there have been many reports interpreting the phylogenetic relationships of *Astragalus*, and even Fabaceae, from a different perspective [7, 8, 13]. It also suggests that the traditional classification of Fabaceae into three subfamilies, which was solely based on morphological traits, is inadequately considered. Although a taxonomic revision is urgently needed, progress in this area has been hindered by the lack of sufficient molecular data. A more robust phylogenetic tree was constructed by integrating *matK* gene sequences from cp. genomes with morphological and chromosomal characteristics [79]. Consistent with the proposition by Marazzi and Sanderson that *Cercis* is monophyletic, *C. canadensis* forms a distinct, independent clade separate from other species, indicating earlier differentiation in the evolution of Fabaceae [80]. However, the number of mitochondrial and chloroplast PCGs used is limited, which may affect the resolution of closely related species, and the low substitution rate and potential recombination of plant mitochondria may lead to slight deviations in phylogenetic inference.

In contrast to the relatively abundant cp. genome data, only two mitochondrial genome sequences of *Astragalus* species are publicly available to date. Among the studied *Astragalus* species, *A. laxmannii* and *A. cicer*—both belonging to the subgenus Hypoglottis—exhibited the closest phylogenetic relationship and formed an independent clade. This result was consistent with findings derived from the concatenated dataset of 65 chloroplast protein-coding sequences (CDSs). Additionally, *A. complanatus* was identified as the first diverging lineage within the *Astragalus* clade [10]. Furthermore, differences in mitochondrial genome synteny among distinct species provided additional evidence supporting their phylogenetic relationships. These findings were congruent with previous studies from multiple perspectives [10, 71], confirming that mitochondrial genomes are another reliable tool for advancing phylogenetic research on *Astragalus*, laying a critical genomic foundation for resolving the evolutionary relationships within this ecologically and economically important genus.

Most *Astragalus* species are nitrogen-fixing plants that establish symbiotic relationships with *rhizobium* to fix atmospheric nitrogen, which is then converted into plant-available nitrogenous compounds This process significantly enhances soil nitrogen content and fertility [81]. Moreover, most species possess stress tolerance traits, including drought resistance, salt-alkali tolerance, and barren tolerance, enabling them to thrive in harsh habitats such as degraded grasslands, saline-alkali lands, and mine reclamation areas [82–84]. Despite widespread academic interest in the evolutionary processes and conservation of *Astragalus*, detailed genomic studies on related species remain scarce. This knowledge gap has impeded in-depth investigations into the intrinsic mechanisms underlying the diversification patterns, endemic characteristics, and causes of rarity in this taxonomic group [85]. This study interprets the genetic variation characteristics and conducts phylogenomic analysis of *Astragalus* from the perspective of mitochondrial genomes, aiming to provide theoretical support for the efficient protection and rational utilization of *Astragalus* resources, the exploration and exploitation of closely related species, and the maintenance of wild population diversity. Meanwhile, it facilitates the full exertion of their ecological governance value, and lays a foundation for maximizing the excavation of other potential values such as medicinal value in the future. Critically, the high-quality mitochondrial genomic resources generated in this study fill the gap in *Astragalus* mitogenome data, providing a powerful molecular tool for dissecting the genetic basis of nitrogen fixation, stress tolerance, and adaptive evolution in this genus, and supporting the development of molecular breeding strategies for *Astragalus* and related forage legumes.

## Conclusion

By integrating three newly sequenced and annotated *Astragalus* species with two previously published ones, this study systematically investigated mitochondrial genome architecture, the characteristics of simple sequence repeats (SSRs) and dispersed repeats, relative synonymous codon usage (RSCU), RNA editing profiles, chloroplast-to-mitochondrion gene transfer events, nucleic acid polymorphism, and phylogenetic relationships of the genus. Our findings revealed key features of *Astragalus* mitochondrial genomes and their evolutionary dynamics: the three novel circular mitochondrial genomes spanned 269,091–364,431 bp and encoded 55–59 genes, with 46 conserved genes shared across all five studied species. Gene loss events, driven by redundancy or horizontal transfer, emerged as a major contributor to mitochondrial genome size divergence among species. SSR analysis indicated tetramers as the dominant repeat type, whereas dispersed repeat abundance exhibited significant interspecific variation. The *nad4* gene harbored the highest number of RNA-editing sites across the five species, and nine specific genes were subjected to negative selection, suggesting their functional constraints in mitochondrial metabolism. Notably, the high nucleic acid variability of *cox2*, *rps4*, *atp8*, and rps12 validated their potential as reliable molecular markers. These markers are not only applicable for the accurate identification of closely related *Astragalus* species but also hold promise for dissecting population genetic structure, quantifying interpopulation gene flow, and assessing the impact of geographic isolation on lineage differentiation. Phylogenetic reconstruction with 41 Fabaceae species clarified the evolutionary placement of the five *Astragalus* species: *A. cicer* and *A. laxmanii* formed a clade closely related to *A. melilotoides*, while *A. complanatus* represented the earliest-diverging lineage, providing robust phylogenetic evidence for interspecific relationships within the genus. This comprehensive analysis illuminates the intricate patterns of mitochondrial genome variation in *Astragalus*. Beyond characterizing genomic features, these results offer novel insights into the taxonomic classification and evolutionary history of *Astragalus* species, and contribute to a broader understanding of mitochondrial genome evolution across the Fabaceae family. From an applied perspective, the identified molecular markers and phylogenetic framework lay a theoretical foundation for the conservation of wild *Astragalus* germplasm resources, the rational utilization of medicinal and ecological species, and the sustainable management of Fabaceae plant populations in natural ecosystems.

## Supplementary Information

Supplementary Material 1.

## Data Availability

The raw data of second and third-generation sequencing of *A. cicer* and *A.* laxmannii were deposited in NCBI (https://www.ncbi.nlm.nih.gov/) under the accession numbers SRR34934999, SRR34934998, SRR34934997 and SRR34934996，respectively. The second-generation sequencing of mitochondrial genomes of *A. melilotoides* was deposited in NCBI (https://www.ncbi.nlm.nih.gov/) under the accession number SRR13870430. The GenBank accessions of mitochondrial genomes of *A. melilotoides* ， *A.cicer and A. laxmannii* was OP279714, PV423826, PQ043269. The GenBank accessions of Chloroplast genomes of *A.cicer* and *A. laxmannii* was PV423827 and PV423828.
